# Advances in Gene Therapy Techniques to Treat *LRRK2* Gene Mutation

**DOI:** 10.3390/biom12121814

**Published:** 2022-12-05

**Authors:** Sun-Ku Chung, Seo-Young Lee

**Affiliations:** Division of KM Science Research, Korea Institute of Oriental Medicine, 1672 Yuseong-daero, Yuseong-gu, Daejeon 34054, Republic of Korea

**Keywords:** *LRRK2* G2019S, zinc finger nucleases, helper-dependent adenoviral vector, CRISPR/Cas9-HDR, adenine base editor, bacterial artificial chromosome-based homologous recombination

## Abstract

Leucine-rich repeat kinase 2 (*LRRK2*) gene mutation is an autosomal dominant mutation associated with Parkinson’s disease (PD). Among *LRRK2* gene mutations, the *LRRK2* G2019S mutation is frequently involved in PD onset. Currently, diverse gene correction tools such as zinc finger nucleases (ZFNs), helper-dependent adenoviral vector (HDAdV), the bacterial artificial chromosome-based homologous recombination (BAC-based HR) system, and CRISPR/Cas9-homology-directed repair (HDR) or adenine base editor (ABE) are used in genome editing. Gene correction of the *LRRK2* G2019S mutation has been applied whenever new gene therapy tools emerge, being mainly applied to induced pluripotent stem cells (*LRRK2* G2019S-mutant iPSCs). Here, we comprehensively introduce the principles and methods of each programmable nuclease such as ZFN, CRISPR/Cas9-HDR or ABE applied to *LRRK2* G2019S, as well as those of HDAdV or BAC-based HR systems used as nonprogrammable nuclease systems.

## 1. Introduction

Parkinson’s disease (PD) is the second most common neurological disorder after Alzheimer’s disease [1,2,3]. Most PD cases occur sporadically in individuals without genetic risk factors. At least 5% of patients with familial PD carry a mutation in one of the several genes, including α-synuclein; *PARK1*, LRRK2; *PARK8*, parkin (PRKN; *PARK2*), PTEN-induced putative kinase 1 (PINK1; *PARK6*), and DJ-1 (*PARK7*). The functional loss or gain of these genes results in the progressive depletion of dopaminergic neurons in the substantia nigra, pathologically inducing cell death. Therefore, in the case of PD caused by genetic factors, a therapeutic effect is expected by fundamentally eliminating genetic mutations. The gene therapy strategy may differ depending on whether it is a functional loss or gain caused by a gene mutation. While loss-of-function mutations in genes encoding parkin, PINK1, and DJ-1 could be conceptually complemented by an exogenous vector expressing the normal gene, the functional gain of monogenic mutations in the genes encoding LRRK2 or α-synuclein should be corrected to alleviate the dominant effect, wherein the mutated base must be replaced with the normal base. In this study, we describe the therapeutic strategies for PD associated with mutations in the *LRRK2* gene.

The *LRRK2* gene belongs to a leucine-rich repeat kinase family that encodes a protein containing an armadillo repeats region, ankyrin repeat region, a leucine-rich repeat domain, a kinase domain, a RAS domain, a GTPase domain, and a WD40 domain [4]. LRRK2 is known to be involved in physiological functions in neurons, such as neurite outgrowth, cytoskeletal maintenance, vesicle trafficking, and autophagic protein degradation. In carrying out these functions, a mutation in exon 41, which encodes the kinase domain of LRRK2, is thought to enhance the kinase activity of LRRK2, leading to neurological disorders such as PD [4,5].

*LRRK2* gene mutations were first identified in 2004 as an autosomal dominant mutation in familial PD [6,7]. Among *LRRK2* mutations, including R114G/C/H, Y1699, and I2020T, the *LRRK2* G2019S mutation is particularly prevalent, with a frequency of 5–6% in familial PD [8,9,10,11,12]. Moreover, in sporadic PD, a high frequency of *LRRK2* G2019S mutation (>18%) was found in some populations, such as the Ashkenazi Jews [13]. Currently, gene replacement therapy for *LRRK2* G2019S with high mutation frequencies has been introduced using various gene therapy tools such as zinc finger nuclease (ZFN), helper-dependent adenoviral vector (HDAdV), and Adenine base editor (ABE) [1,14,15,16].

In addition to these gene editing tools, BAC-based HR can also be used effectively as a gene replacement tool. BAC-based HR can target genes via homologous recombination using BAC DNA [17,18,19,20]. Previously, we reported the potential value of a BAC-based HR system by genetically correcting the *LRRK2* G2019S mutation [21]. As mentioned above, the *LRRK2* G2019S mutation has been treated using various gene-editing tools [1,14,16,21]. This implies the importance of the *LRRK2* G2019S mutation in relation to PD; further, the emergence of various gene correction tools applied to LRRK2 gene correction is also worth noting. Therefore, herein, we comprehensively review the principles and methods of the various gene therapy tools applied to treat the *LRRK2* G2019S mutation.

## 2. Zinc Finger Nuclease (ZFN)-Mediated *LRRK2* Gene Targeting

**Figure 1:**
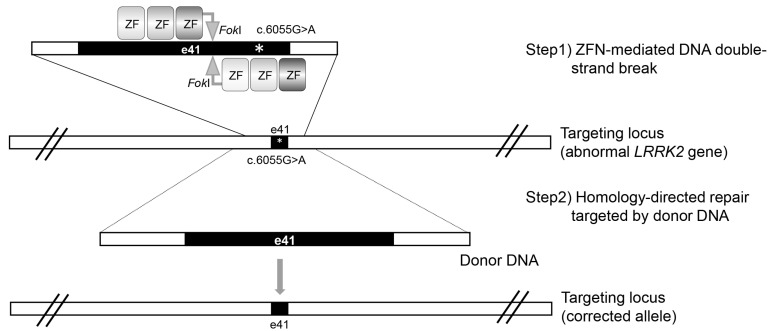
ZFN-mediated *LRRK2* gene correction. Following the induction of the ZFN-mediated DNA double-strand break adjacent to the *LRRK2* G2019S gene mutation, the homology-directed repair was targeted by donor DNA. The “e41” means exon 41 of *LRRK2* gene. The asterisks indicate *LRRK2* c.6055G>A.

ZFN comprises three to six zinc finger motifs that bind DNA, and the type II restriction enzyme *Fok*I cleaves DNA. A pair of ZFNs is used to target non-palindromic DNA sequences, with the ‘left’ ZFN engineered to bind the DNA before the desired cleavage site and the ‘right’ ZFN engineered to bind the DNA after in the opposite orientation. The short spacer sequences between the left and right ZFNs provide DNA cleavage sites for dimerized *Fok*I endonucleases, which are linked to the C-terminus of each ZFN (Figure 1, step1). ZFN specifically cleaves the target DNA sites, which facilitates homologous recombination or non-homologous end-joining events, such as DNA repair. In this process, adding exogenous donor templates can enhance the probability of a homologous recombination event.

By applying linearized targeting DNA as a correction vector and ZFN as a gene scissor, Reinhardt et al. [1] genetically corrected hiPSCs derived from PD fibroblasts with *LRRK2* G2019S mutation. Despite no detailed description of the donor vector or ZFN, ZFN was designed to induce a DNA double-strand break (DSB) adjacent to the *LRRK2* gene mutation (Figure 1, step1). In the DNA repair triggered by a ZFN-mediated DSB, a correction vector is provided as a donor DNA for homologous recombination (Figure 1, step2).

**Figure 2:**
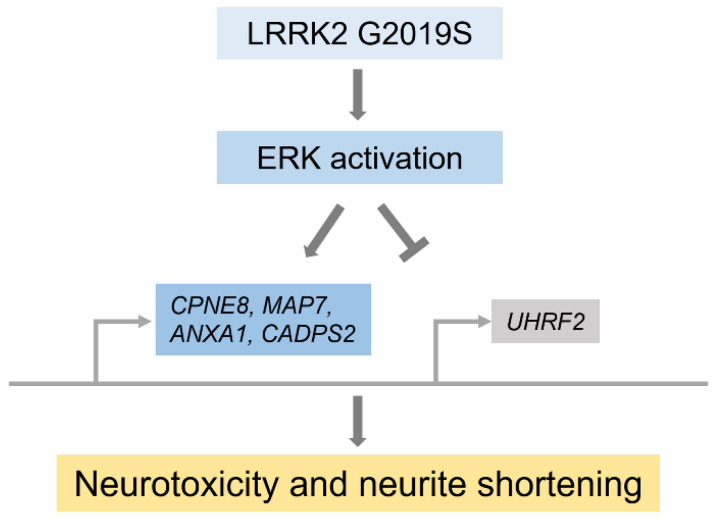
Schematic diagram of gene expression change following ERK activation by LRRK2 G2019S, leading to neurodegeneration [1].

Gene correction phenotypically alleviates the degeneration of the midbrain dopaminergic neurons, including the neurite shortening and apoptotic sensitivity to neurotoxins caused by *LRRK2* G2019S mutation [22,23,24]. Additionally, it reduces the number of autophagosomes, indicating that *LRRK2* G2019S-induced aberrant autophagy [3,23]. The expression of tau and α-synuclein, which affect PD pathogenesis, was also reduced in the gene-corrected cell line; in contrast, the expression of these proteins was high in cells with *LRRK2* G2019S mutation. The tau induces cytotoxicity via aggregation through interactions with α-synuclein. Thus, the increased expression of the two proteins by *LRRK2* G2019S mutation can synergistically affect PD progression [25]. Apart from investigating the expression of these two proteins in the mutant cell line, in comparison with their expression in the isogenic line, Reinhardt et al. recently identified that some genes, including *CPNE8*, *ANXA1*, *MAP7*, *CADPS2*, and *UHRF2*, were dysregulated by the *LRRK2* G2019S mutation, compared with their function in the isogenic control. Gene knockdown experiments confirmed that several genes, including *CPNE8*, *CADPS2*, and *MAP7*, play a role in the cytotoxicity of the *LRRK2* G2019S mutation. The *CPNE8* gene encodes the copine 8 protein, a calcium-dependent membrane-binding protein that plays a role at the interface between the cell membrane and cytoplasm. So far, the association of the gene with PD is unknown, but it is thought that the upregulation of the *CPNE8* gene may additionally affect dendrite shortening or mitophagy due to calcium imbalance directly induced by LRRK2 G2019 [26]. The *CADPS2* gene is known to encode a calcium-dependent activator of the secretion protein family. Although the relevance of this gene to PD is unknown, a unique mutation, CADPS2 p.V559L, is known to cause neurodegeneration with a Lewy body-like pathology [27]. In addition, MAP7 consists of a phosphorylation domain, and two coiled-coil regions capable of interacting with microtubules and kinesin 1, respectively. The expression of MAP7 has the effect of suppressing axon growth and branch formation. In this context, overexpression of MAP7 by LRRK2 G2019S may affect the shortened length of neurite outgrowth in LRRK2 G2019S dopaminergic (mDA) neurons [28]. *ANXA1* gene encodes the anti-inflammatory mediator annexin A1 that exerts neuroprotective effects by contributing to various intracellular membrane trafficking steps in the peripheral nervous system [29]. The upregulation in *ANXA1* gene expression by LRRK2 G2019S seems contradictory in terms of the neuroprotective effect of annexin A1, but transient knockdown of *ANXA1* gene expression under reactive oxygen species still induces dopaminergic neuronal cell death [1]. Although this has limitations in overcoming the apoptotic properties of mDA neurons, it may suggest that the increased expression of annexin A1 by LRRK2 G2019S may rather be a unique pathway related to the survival of mDA neurons. UHRF2 is a kind of E3 ubiquitin ligase that regulates cell proliferation [30]. Unlike the four genes abovementioned, the expression of *UHRF2* in LRRK2 G2019S is downregulated. The downregulation of *UHRF2* gene expression can inhibit the function of the gene involved in cell growth, thus affecting the growth of mDA neurons. In common, *UHRF2*, *CPNE8*, and *CADPS2* were related to the ERK signaling pathway activated by *LRRK2* G2019S. According to a previous report, the *LRRK2* G2019S mutation affects neurite shortening via ERK activity (Figure 2) [1]. Altogether, it could be assumed that the dysregulation of *CPNE8*, *CADPS2*, or *UHRF2* contributes to PD progression via ERK activation induced by the *LRRK2* G2019S mutation.

## 3. Helper-Dependent Adenoviral Vector (HDAdV)-Mediated *LRRK2* Gene Targeting

**Figure 3:**
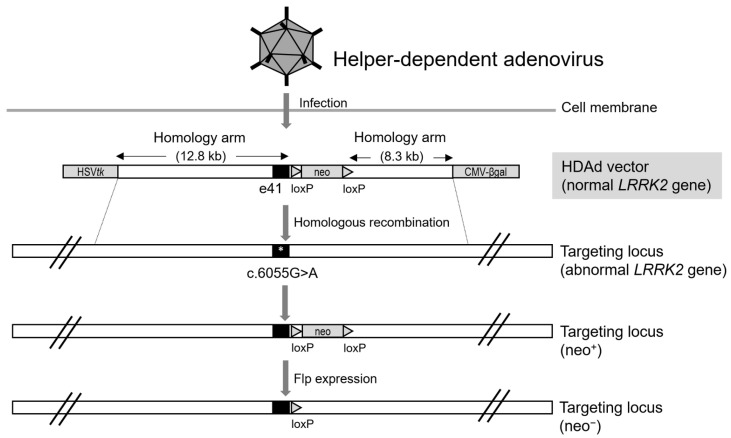
HDAd vector-mediated *LRRK2* gene correction. The targeting of the HDAd vector adjacent to the *LRRK2* gene mutation, *LRRK2* c.6055G>A by homologous recombination.

Adenoviral vectors are widely used as gene-delivery systems for transgene expression. However, to eliminate the adaptive cellular immune response triggered by viral genes and to insert the transgene into the viral vector, most of the essential viral genes, excluding those which are required for encapsidation and DNA replication, are removed and replaced with a cassette that includes the transgene. Deleting these viral genes makes it possible for the modified viral vectors to reproduce on their own; thus, it becomes possible to either replicate the target DNA or package the viral genome depending on the helper virus to propagate the viral vectors, which are referred to as HDAdVs. HDAdVs can be replaced with a large transgene corresponding to approximately 36 kb viral genes removed from the adenoviral vector. As a gene therapy tool, a length of approximately 36 kb as the cloning capacity of HDAdV, which is provided by therapeutic gene constructs, could facilitate gene correction of the target locus through homologous recombination. As a non-integrating vector, HDAdV intrinsically has the property that exogenous genes are not feasibly integrated into target loci, but long homology arms enable HDAdV-mediated gene transfer via homologous recombination (Figure 3).

The nuclear envelope not only structurally serves as a barrier between the nucleus and cytosol but also maintains nuclear stability or chromatin structure. Its impairment fundamentally disrupts cellular homeostasis, leading to various diseases and aging. Interestingly, Liu et al. reported a correlation between nuclear architecture impairment and PD etiology by studying a genetic isogenic PD model containing the *LRRK2* G2019S mutation treated with an HDAdV-mediated gene correction system. The authors found that the *LRRK2* G2019S mutation affects PD onset through serial passages to mimic cellular aging. In late-passage cells (at least 14 passages), nuclear structural aberrations were observed, and the activity of lamin B1 and B2 located in the inner nuclear membrane was abnormally increased by the *LRRK2* G2019S mutation, which could impair the nuclear envelope. Additionally, the *LRRK2* G2019S mutation revealed epigenetic abnormalities appearing in senescent cells. Compared with the observations in normal NSCs, H3K4me3, an epigenetic marker associated with neural aging, was enriched in genes involved in neurogenesis or neural function, indicating that *LRRK2* G2019S affects the clonogenicity or neural differentiation of stem cells toward MAP2- or Tuj1-positive cells.

## 4. Bacterial Artificial Chromosome (BAC)-Mediated *LRRK2* Gene Targeting

BAC DNA has also been used to generate genetically engineered organisms. Homologous recombination is the principle of gene targeting by BAC DNA. To efficiently insert exogenous BAC DNA into a target locus via homologous recombination, a large homology sequence of 100 kb or more is required. Thus, homologous recombination efficiency depends on homology arm length; the homologous recombination efficiency is low when the homology arm length is less than 80 kb [17]. Indeed, by applying naïve BAC DNA with intact homology arms of over 150 kb, we were able to efficiently produce gene-modified human embryonic stem cells [17,18,19,20]. The linearization of BAC DNA by a unique restriction endonuclease contained in the BAC DNA vector mimics the effect of DNA DSBs, triggering homologous recombination events in cells as a DNA repair system.

**Figure 4:**
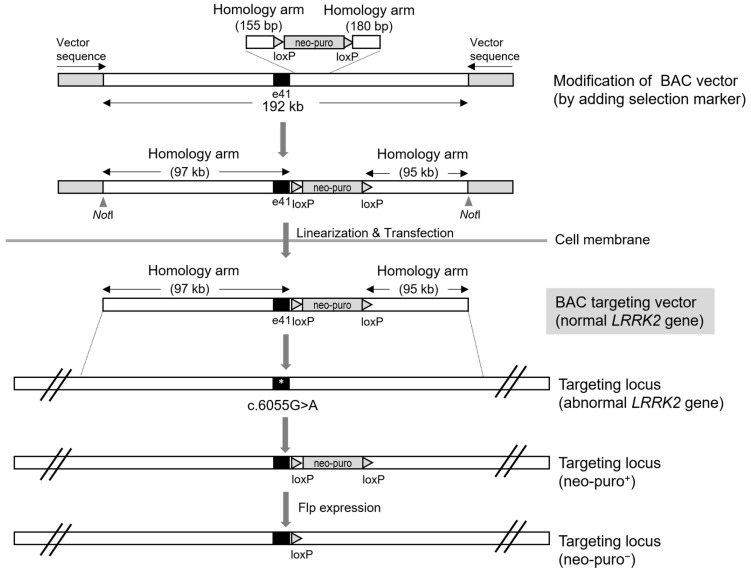
BAC-mediated *LRRK2* gene correction. For efficient screening, naïve BAC DNA was modified by adding selection markers, neomycin, or puromycin. After the modified BAC vector was linearized by the *Not*I restriction endonuclease, it was transfected into the iPSCs. The huge homology arm enhanced the probability of a homologous recombination event at the targeting locus. Finally, the selection markers were eliminated from the floxed allele, expressing the Flp enzyme.

Unmodified BAC DNA does not contain antibiotic resistance marker genes such as puromycin or neomycin as selection markers. Thus, it should be genetically modified prior to its introduction into mammalian cells. Homologous recombination has also been applied to introduce selection markers in intact BAC DNA. However, unlike the case with mammalian cells, a shorter length (at least 50 bp) of the homologous sequence is required for recombination in *Escherichia coli*. Once BAC DNA is introduced into the cells, antibiotic-resistant clones can be acquired during the selection step. By applying a BAC-mediated homologous recombination system, we reported a *LRRK2* gene-corrected clone that had been genetically replaced with BAC DNA harboring the normal *LRRK2* gene (Figure 4). We used BAC DNA with a total sequence length of approximately 200 kb to treat the *LRRK2* G2019S mutation. The sequence length of the homology arm flanking a selection marker was approximately 100 kb, which was efficiently driven into the *LRRK2* G2019S mutation region to target the mutant allele. While investigating isogenic NSC lines derived from iPSCs genetically corrected by BAC DNA, we analyzed the expression of NSC markers, such as SOX1, NESTIN, and PAX6 during NSC differentiation. Particularly, it was confirmed that SOX1, which was partially expressed in the pathogenic line, was completely recovered (in terms of its expression) in the correction line [21].

## 5. CRISPR/Cas9 and PiggyBac-Mediated *LRRK2* Gene Targeting

**Figure 5:**
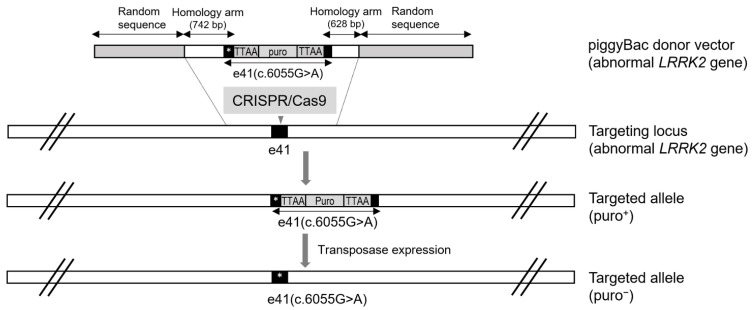
Generation of *LRRK2* G2019S-mutant cell by CRISPR/Cas9 and piggyBac transposon system. In the piggyback donor vector, the TTAA sequence upstream of puro was a silent mutation from TAAA. The selection marker between the TTAA sequences is seamlessly released by transposase.

As a third-generation gene scissor, CRISPR/Cas9 is a representative programmable nuclease used as a gene editing tool together with a first-generation ZFN or a second-generation TALEN (transcription activator-like effector nuclease). Following the guide RNA that recognizes the target site in the genome, Cas9 endonuclease can specifically induce DNA DSBs, which subsequently induces either of two main pathways, namely non-homologous end joining (NHEJ) or the homologous recombination pathway, as a DNA repair mechanism. Compared to homologous recombination, NHEJ events predominantly occur in the repair of DNA DSBs [31], leading to imprecise insertions or deletions adjacent to the target site. Hence, similar to ZFN or TALEN, CRISPR/Cas9 requires a donor template to replace the target sequence via homology-directed repair. Donor templates with diverse sequence lengths, and those of the single-strand and double-strand types were used for homology-directed repair. Without a resistance-dependent selection step, cumbersome procedures, such as serial dilution to single cells, expansion from single cells, and genotyping of clones, are required to identify whether the donor DNA is precisely integrated into the target site. Hence, donor DNA with selection markers would facilitate the identification of gene-edited clones, thereby saving time, labor, and cost throughout the screening. Finally, once the Gene-edited clones are identified, the selection marker of the donor DNA is usually removed using the Cre/LoxP or Flp/Frt recombination system. However, even if the selection marker is removed, recombination sites, such as the 34-bp LoxP or Frt site sequences remain in the genome. These sequences have caused serious problems in transgenic mice produced by genetic recombination for decades, but the PiggyBac Transposon System can seamlessly remove these sequences (Figure 5).

Although not attempting to investigate a gene correction strategy, Qing et al. reported a footprint-free *LRRK2* G2019S isogenic line genetically produced with CRISPR/Cas9 and the PiggyBac Transposon System [15]. Consistent with previous reports [23,32], they reaffirmed that the *LRRK2* G2019S mutation significantly affects the neurite length and a few neurite branches in dopaminergic neurons differentiated from the isogenic line. Additionally, they identified positive Ser129 phosphorylation of the alpha-synuclein in dopaminergic neurons with neurite lengths greater than 2000 µm, implying that alpha-synuclein may play an important role in forming or maintaining long neurites. Two effects identified by *LRRK2* gene mutation, long neurite length or phosphorylated alpha-synuclein, were similar to those of treatment with the *LRRK2* kinase inhibitor (IN-1) [24].

## 6. Adenine Base Editor (ABE)-Mediated *LRRK2* Gene Targeting

**Figure 6:**
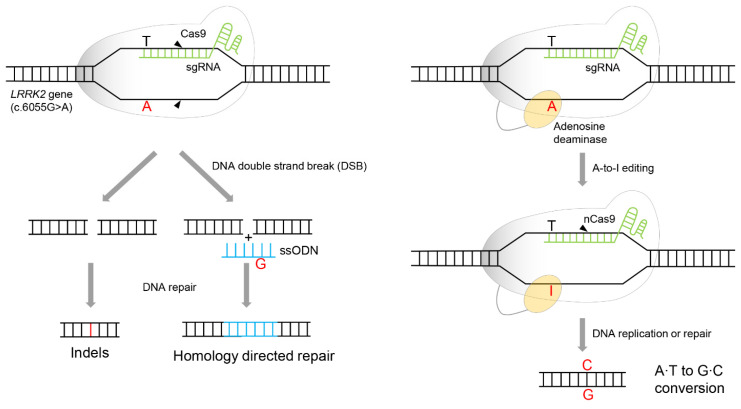
ABE-mediated *LRRK2* gene targeting compared to CRISPR/Cas9. CRISPR/Cas9-homology-directed repair (**left**) and ABE system (**right**). nCas9 means Cas9 nickase harboring D10A mutation. sgRNA indicates single guide RNA, including a targeting sequence and a Cas9 or adenosine deaminase-recruiting sequence. ssODN means single-stranded oligodeoxynucleotide served as DNA donor template. A; adenosine, T; thymidine, G; guanosine, C; cytidine, I; inosine.

As previously mentioned, CRISPR/Cas9 induces DSB. This means that indels (insertions and deletions) are induced on alleles other than the target allele. Although mutations within the target DNA are treated by HDR, additional unintended mutations in other alleles may occur. According to Chang et al., in exon41, where c.G6055A encoding LRRK2 G2019S is located, nearly 60% of indels were generated by CRISPR/Cas9 [16]. Although the correction effect of HDR is expected to be through DNA damage induced by CRISPR/Cas9, the CRISPR/Cas9’s off-target effects reduce gene editing efficiency by inducing additional scars near the target DNA (Figure 6, left). To solve this problem, they applied a gene-editing tool that can selectively treat specific mutation sites, c.G6055A. This indicates that guanine selectively replaces the adenine of G6055A, preferably without inducible mutations other than the target mutation.

An ABE is a system that selectively edits adenine bases. It comprises dCas9 fused with TadA, a dimeric tRNA adenine deaminase derived from *Escherichia coli*. It has evolved to efficiently deaminate adenine in the target DNA [33]. dCas9 was substituted with an amino acid (D10A) to nick the complementary strand of the mutation site [34,35], instead of abolishing nuclease activity to avoid inducing DSB. It converts T-A to T-I by deaminating adenosine to inosine in the target DNA. dCas9 nicks the complementary strand of the mutation site. DNA repair/replication converts T-I to C-G by recognizing inosine as guanosine [36]. ABE’s specificity improved the correction efficiency of *LRRK2* G2019S mutation and minimized DNA damage by modifying Cas9 to dCas9 (Figure 6, right). Moreover, the indel effect, which occurs frequently in CRISPR/Cas9, did not occur at all. However, ABE recognizes adenines present in the vicinity, other than adenine mutations, and induces additional missense mutations. Nevertheless, the correction efficiency was eventually improved by approximately fourfold compared to that of conventional CRISPR/Cas9 [16].

In *LRRK2* gene-corrected clones, α-synuclein phosphorylation and kinase activity by autophosphorylation of LRRK2 in the LRRK2 G2019S mutant cells was reduced. As the factors affecting dopaminergic neural degeneration disappeared, the phenotypes, such as lengthening of the shortened neurites or the absence of apoptosis due to a decrease in caspase 3 activity, were also improved [16].

## 7. Conclusions

Recently, Schweitzer et al. successfully treated Parkinson’s disease by implanting personalized iPSC-derived dopamine progenitor cells into the patient’s putamen. Although the patient has idiopathic Parkinson’s disease, the genetic mutation is unknown, an innovative treatment strategy was presented in which autologous cell therapy was successful based on iPSCs prepared from patient-derived skin cells. Even in patients with Parkinson’s disease-related gene mutation, if the related genes are treated based on the various gene therapies introduced so far, these gene-corrected cells can provide resources for autologous cell therapy [37].

As already mentioned in the introduction, genetic mutations associated with Parkinson’s disease can be divided into autosomal recessive mutations and autosomal dominant mutations. Delivery using a viral vector is very useful for treating genes with autosomal recessive mutations. LeWitt et al. have successfully treated the GAD gene, encoding glutamic acid decarboxylase, which is the rate-limiting enzyme for GABA production, using an adeno-associated viral vector to restore GABA production [38]. In this way, when the function of a gene is decreased due to mutation or an unknown cause, a therapeutic strategy for restoring the function of a gene through a viral vector will be possible. However, in the case of phenotypic dysregulation caused by autosomal dominant mutations such as *LRRK2* or α-synuclein gene, the problematic mutation region should be fundamentally removed or replaced. Viral vectors are efficient in gene delivery, but they may require more sophisticated gene replacement strategies to treat autosomal dominant mutations.

Considering that LRRK2 activity is increased not only in *LRRK2* mutant PD but also in idiopathic PD [39], therapies involving LRRK2 inhibition are promising as a PD treatment strategy and are underway in clinical trials [40]. One of the autosomal dominant mutations in the *LRRK2* gene, the *LRRK2* G2019S mutation, is frequently found as a hot spot mutation in sporadic and familial cases of PD [41]. Considering the frequency of this gene mutation, it provides a representative case for a PD gene therapy strategy. To date, gene therapies targeting the *LRRK2* G2019S mutation have been attempted, from first-generation gene scissors such as ZFN to third-generation gene scissors such as the CRISPR/Cas9 system. Furthermore, the CRISPR/Cas9 system was further advanced and combined with an adenine base editor, an editing tool that can replace specific adenine nucleotide sequences. Apart from these programmable nuclease-based gene therapy tools, various other non-programmable gene therapy systems, such as HDAdV or BAC-based HR systems, have been established to successfully treat the *LRRK2* G2019S mutation.

Several gene mutations associated with PD are worthy of consideration as targets for therapeutic intervention, but interestingly, the latest gene therapies have applied the same target to *LRRK2*, particularly the *LRRK2* G2019S mutation. Among the latest gene therapies, gene therapy strategies, such as ZFN, CRISPR, and CRISPR base editors, have been commonly applied for treating genetic diseases including Duchenne muscular dystrophy, Fanconi anemia, beta-thalassemia, et al., and diseases arising from *LRRK2* G2019S mutation [1,16,42,43,44]. As nonprogrammable nuclease systems, the advent of HDAdV and BAC-based HR systems, which has not been frequently attempted for any other diseases, was additionally attempted for the *LRRK2* G2019D mutation, thereby broadening the choice of gene therapy strategies [14,21]. Therefore, gene therapy strategies for *LRRK2* G2019S mutation can be used as a valuable reference to treat other genetic diseases. *LRRK2* gene-corrected cells based on these therapeutic tools provide an isogenic line model for studying the pathobiological effects of *LRRK2* mutations. To generate valuable therapeutic resources, the development of gene therapy tools for the *LRRK2* G2019S mutation has progressed in the direction of defect minimization. In other words, gene therapy tools have evolved with scarless genome editing, increasing their accuracy without off-target issues. Therefore, various genetic tools for treating *LRRK2* G2019S mutations, such as ZFN, HDAdV, BAC-based HR, CRISPR/Cas9, and ABE, could suggest therapeutic strategies for other genetic diseases as well.

## Data Availability

Not applicable.
